# Higher mortality and hospital charges in patients with cirrhosis and acute respiratory illness: a population-based study

**DOI:** 10.1038/s41598-018-28317-w

**Published:** 2018-07-02

**Authors:** Biyao Zou, Yee Hui Yeo, Donghak Jeong, Edward Sheen, Haesuk Park, Pauline Nguyen, Yao-Chun Hsu, Gabriel Garcia, Mindie H. Nguyen

**Affiliations:** 1 Stanford University Medical Center, Division of Gastroenterology and Hepatology, Palo Alto, CA 94304 USA; 2 University of Florida College of Pharmacy, Pharmaceutical Outcomes & Policy, Gainesville, FL 32608 USA; 3 Fu-Jen Catholic University Hospital, Division of Gastroenterology, New Taipei, Taiwan

## Abstract

Both cirrhosis and acute respiratory illness (ARI) carry substantial disease and financial burden. To compare hospitalized patients with cirrhosis with ARI to cirrhotic patients without ARI, a retrospective cohort study was conducted using the California Office of Statewide Health Planning and Development database. To balance the groups, propensity score matching (PSM) was used. We identified a total of 46,192 cirrhotic patients during the three study periods (14,049, 15,699, and 16,444 patients, respectively). Among patients hospitalized with cirrhosis, the ARI prevalence was higher in older age groups (p < 0.001), the Asian population (p = 0.002), non-Hispanic population (p = 0.001), and among Medicare patients (p < 0.001). Compared to controls, patients with ARI had 53.8% higher adjusted hospital charge ($122,555 vs. $79,685 per patient per admission, *p* < 0.001) and 35.0% higher adjusted in-hospital mortality (*p* < 0.001). Older patients, patients with alcoholic liver disease or liver cancer were at particularly higher risk (adjusted hazard ratio = 2.94 (95% CI: 2.26–3.83), 1.22 (95% CI: 1.02–1.45), and 2.17 (95% CI: 1.76–2.68) respectively, *p* = 0.028 to <0.001). Mortality rates and hospital charges in hospitalized cirrhotic patients with ARI were higher than in cirrhotic controls without ARI. Preventive efforts such as influenza and pneumococcal vaccination, especially in older patients and those with liver cancer, or alcoholic liver disease, would be of value.

## Introduction

Liver cirrhosis is a worldwide challenge^1^. As reported in the Global Burden of Disease 2010 Study, cirrhosis accounted for 31 million disability-adjusted life-years and 2% of all deaths worldwide in 2010^2^. Cirrhosis has also become a major public health concern in the United States (U.S.). The overall prevalence of cirrhosis in the U.S. between 1999 and 2010 was 0.27% based on the National Health and Nutrition Examination Survey, which was approximately 633,000 Americans according to 2010 US census data^3,4^. Cirrhosis was also ranked the twelfth leading cause of death in the U.S. in 2013, accounting for 37,890 deaths^5^. Nonetheless, a recent study demonstrated that liver-related mortality in the U.S. has actually been underestimated over the past two decades^6^.

Cirrhotic patients are more likely to have immune dysfunctions and vulnerable to infections from diverse pathophysiological mechanisms including reticuloendothelial dysfunctions, neutrophil functional impairment, and abnormal immunoglobulin synthesis^7^. As a result, patients with cirrhosis are more prone to infections especially acute respiratory illness (ARI) (influenza, pneumonia, and acute bronchitis/bronchiolitis) which is one of the most common acute infections in hospitalized patients. A recent study found that within a national sample of 270,000 hospitalized patients in 2009, the prevalence of pneumonia and influenza was approximately 0.38% and 0.027%, respectively^8^. The mortality of ARI is also striking. In 2013, 56,832 patients died from pneumonia and influenza in the U.S.^8^. What’s more, seasonal influenza has the highest disease burden on the very young, very old population and the patients with comorbidities, causing 140,000 to 710,000 influenza-related hospitalizations and 12,000 to 56,000 deaths each year^9^. As such, ARI may aggravate the already compromised hepatic reserve of cirrhotic patients and worsen health outcomes^10^.

The economic burden associated with cirrhosis is also significant. In the U.S., the total direct cost of cirrhosis was approximately $2.5 billion in 2004, while the indirect cost was $10.6 billion^11^. Costs from ARI have also been found to be high. The annual direct medical cost of seasonal influenza was $10.19 billion in 2010 based on estimates from the U.S. census bureau^12^. In 2013, more than $19.9 billion was spent on pneumonia and influenza-related care^8^. These high costs, therefore, suggest that preventive care may be of high value in cirrhotic patients. Indeed, previous studies have reported that influenza vaccination could reduce not only the associated mortality from ARI but also the economic burden associated with ARI^7,10^. The influenza and pneumococcal vaccine coverage remained dismally low in the U.S. According to the Center for Disease Control (CDC) surveys, only 38.5% of adults (aged 18 years and older) had received an influenza vaccine^13^. The pneumococcal vaccine coverage on adults aged 19–64 years at increased risk for pneumococcal disease was 23.0%^14^. However, data on influenza and pneumonia vaccination coverage for the cirrhotic population is limited and there have been few recent studies, examining the morbidity, mortality and economic burden associated with ARI in this population.

We, therefore, conducted a population-based study to determine the prevalence of ARI among hospitalized patients with cirrhosis and compare the number of hospitalizations, hospital days, hospital charges, mortality rates, cause of death, and predictors for higher hospital charges and mortality between cirrhotic patients with ARI and cirrhotic controls without ARI in the U.S.

## Results

### Patient characteristics and ARI prevalence

**Figure 1:**
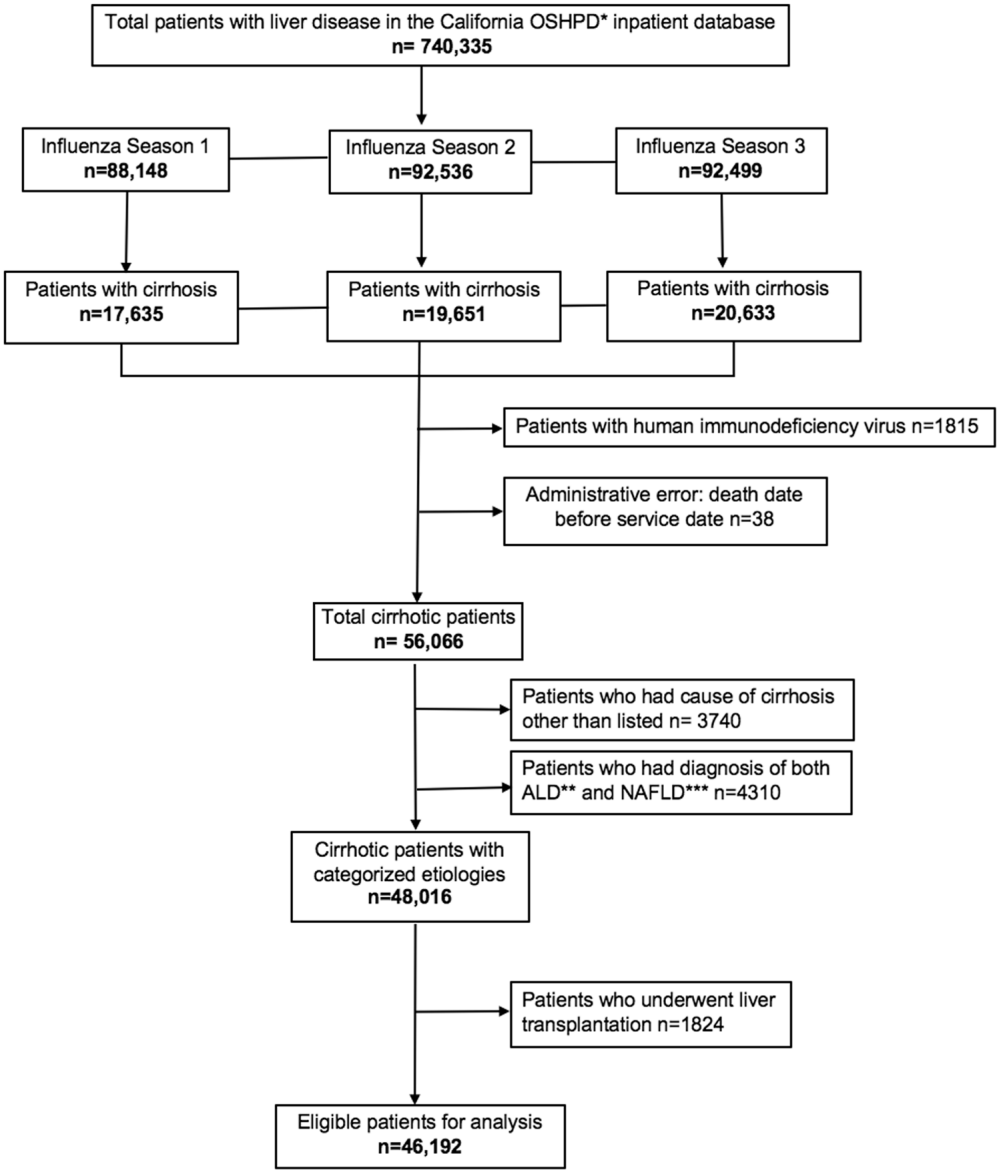
Flow diagram of number of patients included in cirrhosis groups. Influenza season 1: 10/3/2010–5/21/2011. Influenza season 2: 10/2/2011–5/19/2012. Influenza season 3: 9/30/2012–5/18/2013. *Office of Statewide Health Planning and Development. **Alcoholic liver disease. ***Non-alcoholic fatty liver disease.

Figure 1 reports the process of patient selection and enrollment for the study. A total of 46,192 cirrhotic patients were eligible with 14,049 (ARI n = 1240, No ARI n = 12,809), 15,699 (ARI n = 1311, No ARI n = 14,388) and 16,444 patients (ARI n = 1410, No ARI n = 15,034) in the 2010–11, 2011–12 and 2012–13 influenza seasons, respectively.

**Figure 2:**
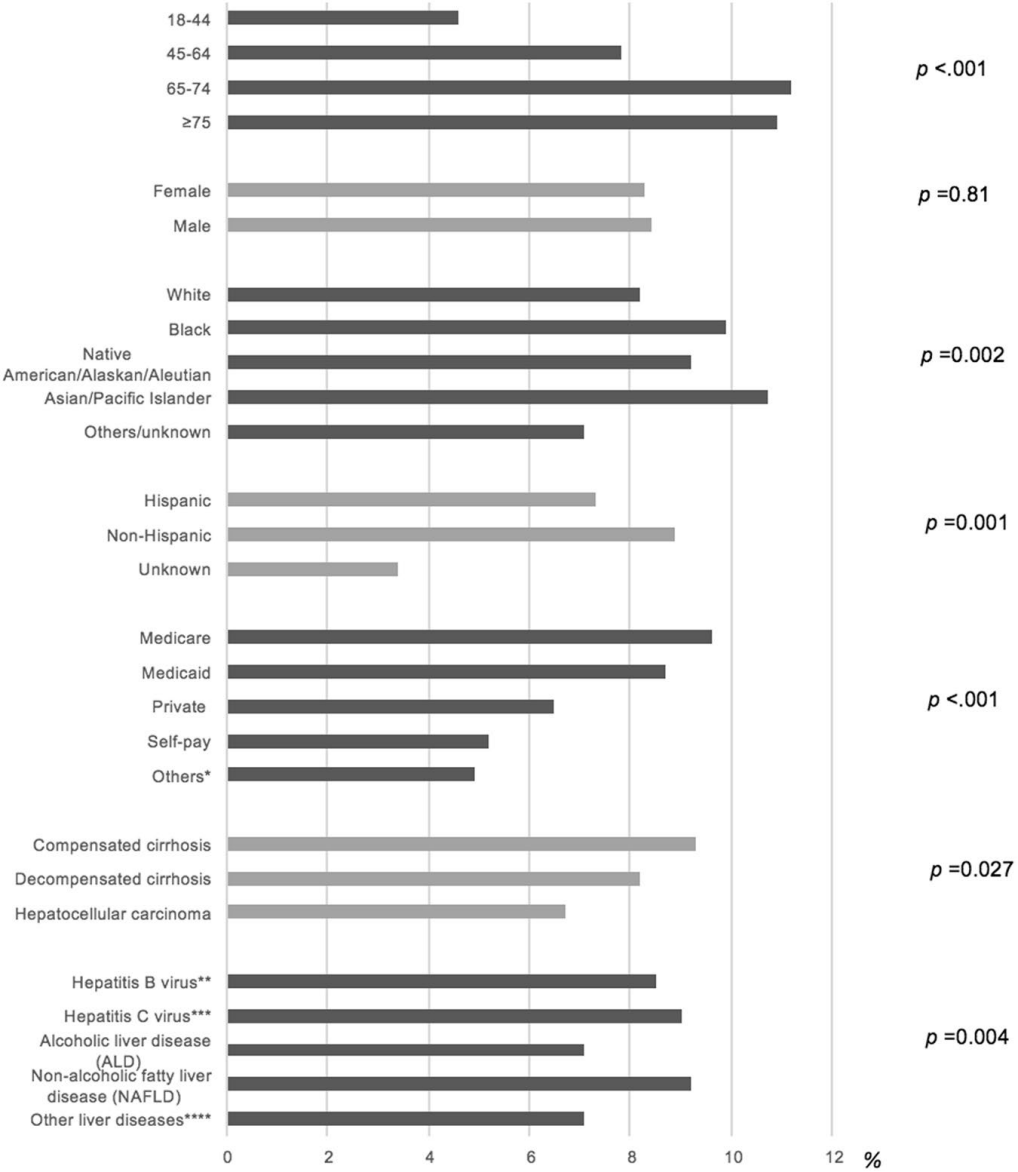
Prevalence of acute respiratory infection in subgroups of cirrhotic patients in the 2011–2012 season. The x axis represents percentage of patients with acute respiratory illness in different subgroups of cirrhotic patients. The y axis shows subgroups of age, sex, race, ethnicity, insurance, severity of cirrhosis, and cause of cirrhosis. The p-values on the right side shows whether the difference of the prevalence within subgroups is significant. *Worker’s compensation, county indigent program, other government, other indigent program, other payer. **HBV patients (with or without HCV, ALD, NAFLD). ***HCV patients (with or without ALD, NAFLD). ****Autoimmune hepatitis, alpha-1-antitrypsin deficiency, hereditary hemochromatosis, hemochromatosis due to repeated red blood cell transfusions, other hemochromatosis, disorders of copper metabolism, cholangitis.

Among patients hospitalized with cirrhosis, the prevalence of ARI was relatively stable across the three influenza seasons: 8.8%, 8.4% and 8.6% in the 2010–11, 2011–12, and 2012–13, respectively. Among cirrhotic patients in the middle period (2011–12 season), the prevalence of ARI was higher in older age groups (*p* < 0.001), the Asian population (*p* = 0.002), non-Hispanic population (*p* = 0.001), and among Medicare patients (*p* < 0.001) (Fig. 2). Consistent findings were demonstrated in the 2010–11 and 2012–13 seasons (Supplementary Table 1).

After propensity score matching, the study population included 1240 and 3004, 1311 and 3240, 1409 and 3382 of ARI and non-ARI patients, respectively in the 2011–12 (Table 1**)**, 2010–11, and 2012–13 (Supplementary Table 2) influenza seasons. No significant differences between cirrhotic patients with and without ARI were found in matched variables (all *p* > 0.05).

**Table 1:** Characteristics of cirrhotic patients with and without acute respiratory illness (ARI) in the 2011–2012 season: after propensity score matching.

Patient number	Cirrhotic patient (n = 4551)			*Standardized difference*
Covariates	ARI	No ARI	*P-*value	
	n = 1311 (%)	n = 3240 (%)		
**Age**				
18–44	75 (5.7)	211 (6.5)	0.73	−1.4
45–64	774 (59.0)	1943 (60.0)		
65–74	290 (22.1)	652 (20.1)		
≥75	172 (13.1)	434 (13.4)		
**Sex**				
Female	498 (38.0)	1230 (38.0)	0.86	−0.7
Male	813 (62.0)	2010 (62.0)		
**Race**				
White	894 (68.2)	2275 (70.2)	0.80	1.0
Black	145 (11.1)	319 (9.9)		
Native American/Alaskan/Aleutian	10 (0.76)	22 (0.68)		
Asian/Pacific Islander	107 (8.2)	219 (6.8)		
Others/unknown	155 (11.8)	405 (12.5)		
**Ethnicity**				
Hispanic	366 (27.9)	921 (28.4)	0.67	−1.6
Non-Hispanic	941 (71.8)	2284 (70.5)		
Unknown	4 (0.31)	35 (1.08)		
**Insurance**				
Medicare	669 (51.0)	1708 (52.7)	0.96	−0.2
Medicaid	407 (31.1)	868 (26.8)		
Private	132 (10.1)	366 (11.3)		
Self-pay	42 (3.2)	109 (3.4)		
Others^a^	61 (4.7)	189 (5.8)		
**Severity of cirrhosis**				
Compensated cirrhosis	283 (21.6)	722 (22.3)	0.63	1.9
Decompensated cirrhosis	975 (74.4)	2373 (73.2)		
Hepatocellular carcinoma (HCC)	53 (4.0)	145 (4.5)		
**Cause of cirrhosis**				
Hepatitis B virus^b^	155 (11.8)	413 (12.8)	0.71	1.4
Hepatitis C virus^c^	712 (54.3)	1731 (53.4)		
Alcoholic liver disease (ALD)	285 (21.7)	699 (21.6)		
Non-alcoholic fatty liver disease (NAFLD)	82 (6.3)	189 (5.8)		
Other liver diseases^d^	77 (5.9)	208 (6.4)		
**Comorbidities**				
Cardiovascular disease	715 (54.5)	1699 (52.4)	0.88	−0.6
Diabetes mellitus	537 (41.0)	1307 (40.3)	0.90	−0.5
Hypertension	876 (66.8)	2160 (66.7)	0.65	−1.7
Any cancer (other than HCC)	107 (8.2)	228 (7.0)	0.32	4.1
Alcohol use/abuse	669 (51.0)	1684 (52.0)	0.85	0.7
Drug abuse	707 (53.9)	1749 (54.0)	0.76	−1.2
Hyperlipidemia	341 (26.0)	821 (25.3)	0.87	0.6
Chronic obstructive pulmonary disease	539 (41.1)	1212 (37.4)	0.79	1.1
Chronic kidney disease	233 (17.8)	536 (16.5)	0.82	0.9
Mental illness	208 (15.9)	507 (15.7)	0.71	1.4
**History of severe comorbidities**				
Renal failure	498 (38.0)	1159 (35.8)	0.87	0.6
Cardiac failure	413 (31.5)	911 (28.1)	0.64	1.9
Major neurologic events	123 (9.4)	294 (9.1)	0.89	0.5
Severe hematologic conditions	343 (26.2)	853 (26.3)	0.96	−0.2
Multi-organ failure	188 (14.3)	406 (12.5)	0.81	1.0
Sepsis	304 (23.2)	712 (22.0)	0.87	−0.7
Hepatic failure	164 (12.5)	392 (12.1)	0.62	1.9
Respiratory failure	303 (23.1)	648 (20.0)	0.70	1.7

^a^Worker’s compensation, county indigent program, other government, other indigent program, other payer.

^b^HBV patients (with or without HCV, ALD, NAFLD).

^c^HCV patients (with or without ALD, NAFLD).

^d^Autoimmune hepatitis, alpha-1-antitrypsin deficiency, hereditary hemochromatosis, hemochromatosis due to repeated red blood cell transfusions, other hemochromatosis, disorders of copper metabolism, cholangitis.

### Hospital Utilization

#### Number of Hospitalizations, Length of Stay, and Hospital Charges

**Figure 3:**
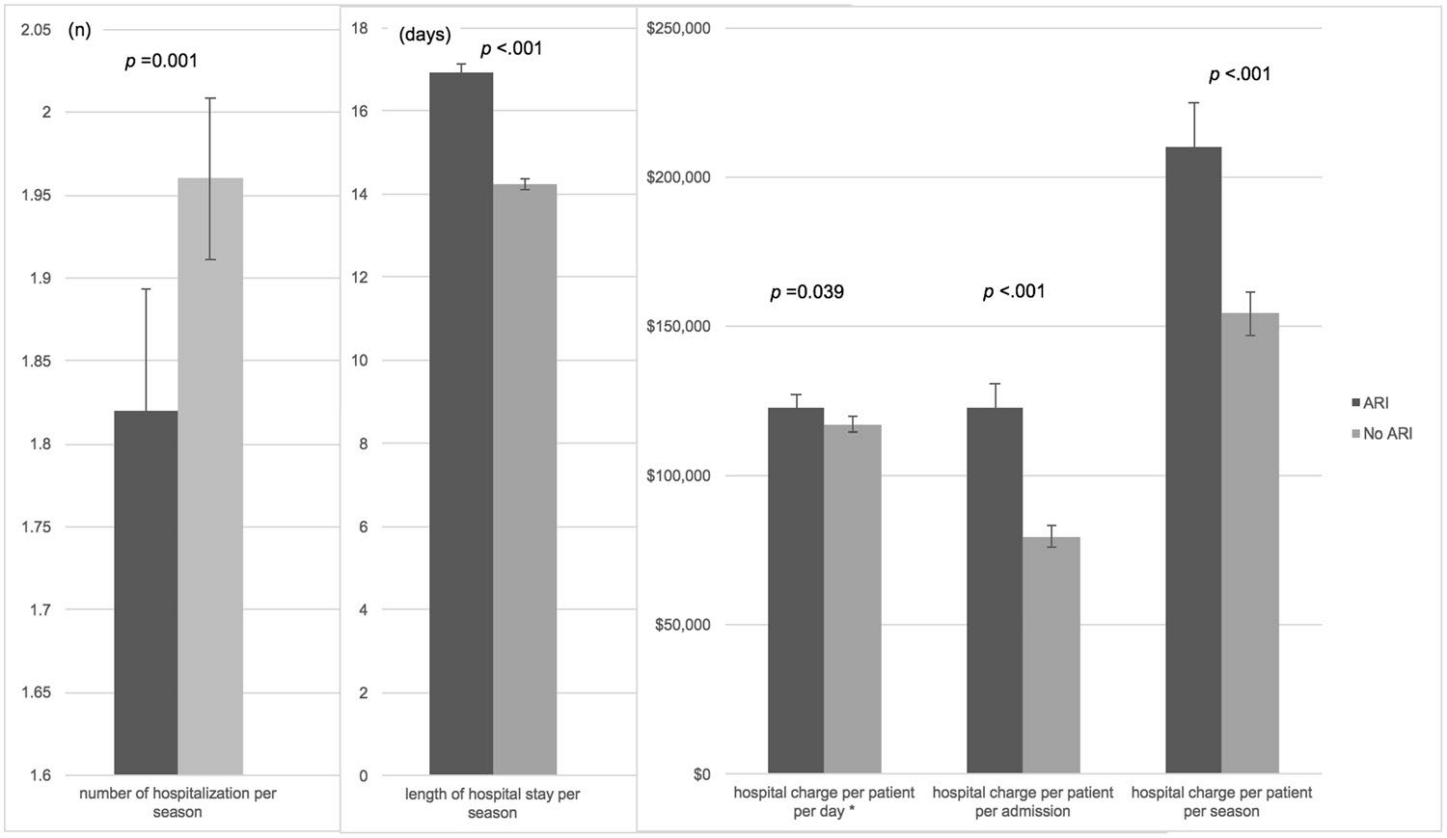
Adjusted number of hospitalization, length of hospital stay, charge per patient per day, hospital charge per patient per admission, and hospital charge per patient per season for cirrhotic patients with and without acute respiratory illness (ARI) in the 2011–2012 season. Results are adjusted for age, sex, race, ethnicity, insurance, severity of cirrhosis, cause of cirrhosis, comorbidities and history of severe comorbidities. The p-values above the bars mean that the difference between cirrhotic patients with and without acute respiratory illness is significant. The black lines on the bars represent the corresponding 95% confidence intervals. *The value of charge on the y axis equals to the *hospital charge per patient per day* times 10.

After PSM and during the 2011–2012 influenza season, cirrhotic ARI patients experienced longer length of hospital stays than in cirrhotic patients without ARI (16.92 vs. 14.23 days, *p* < 0.001) though they had fewer hospital encounters (1.82 vs. 1.96, *p* = 0.001) **(**Fig. 3**)**. The hospital charge per patient per admission for ARI patients was also 53.8% higher than that of patients without ARI ($122,555 vs. $79,685, *p* < 0.001). We also observed a 36.0% higher charge for ARI patients over non-ARI patients in the hospital charge per patient per season ($209,873 vs. $154,288, *p* < 0.001) and a 4.6% higher charge per patient per day ($12,254 vs. $11,717, *p* = 0.039). There were no significant changes in these results across the three study periods for ARI and Non-ARI groups, except for the length of hospital stay per season in ARI group, which showed a slight decrease from 17.27 days in 2010–2011 to 16.72 days in 2012–2013 (*p* = 0.033) (Supplementary Fig. 1).

In the subgroup of patients who required mechanical ventilation, we found that ARI patients requiring mechanical ventilation had more than double (2.22-fold) the seasonal hospital charge per patient than those not requiring ventilation, with total charges of $388,578 vs. $175,370 per patient per season (*p* < 0.001) (Supplementary Fig. 2**)**.

### Mortality

#### Mortality Rates

**Figure 4:**
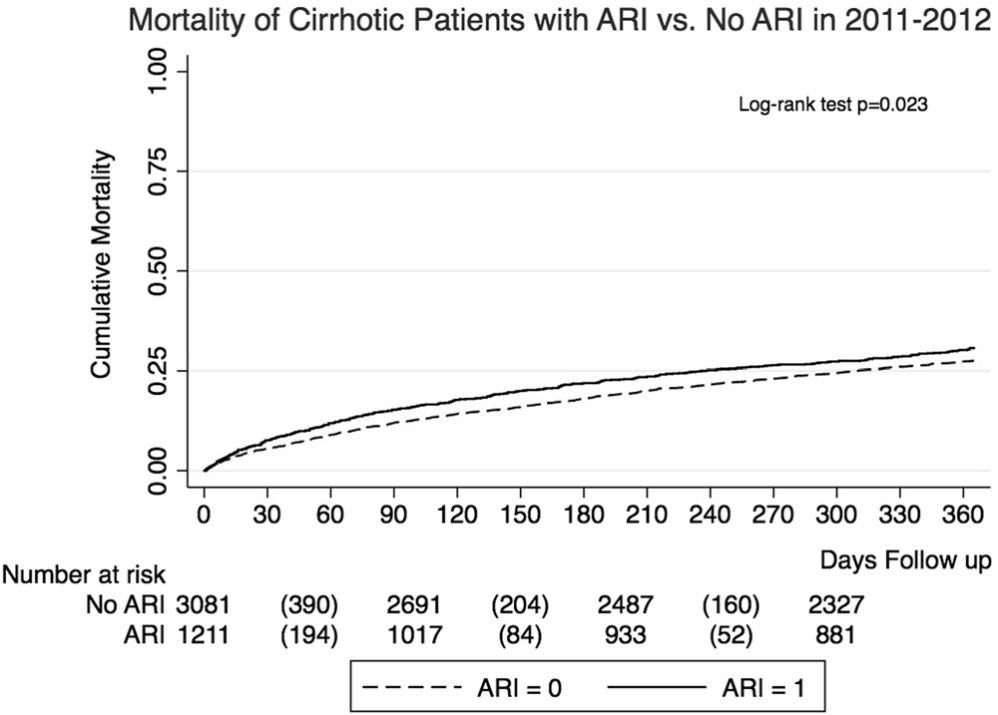
Cumulative mortality for cirrhotic patients with and without acute respiratory illness (ARI) in the 2011–2012 season. The p-value was obtained from the log-rank test, which shows the difference between mortality rates of cirrhotic patients with and without ARI was significant. The number at risk below the x axis describes the number of cirrhotic patients with and without ARI who were at risk of death along the timeline of follow-up.

After PSM, a total of 621 cirrhotic patients who were diagnosed with ARI during the 2011–12 season died before December 21, 2013 while the number of deaths among those without ARI was 1,327. The overall mortality rate among ARI patients was 47.4% while that among non-ARI patients was 41.0%. Specifically, the in-hospital, 30-day and one-year post-discharge morality rates among ARI patients were significantly higher than that among patients without ARI (7.6% vs. 5.8%, *p* < 0.001; 14.6% vs. 11.5%, *p* = 0.023; and 35.9% vs. 31.3%, *p* = 0.001, respectively). As illustrated from the Kaplan-Meier analysis, it was clear that within a follow-up period of one year from the date of hospital discharge, the mortality rate among ARI patients was consistently higher than that among patients without ARI (log rank test, *p* = 0.023) (Fig. 4).

#### Cause of Death

The most common cause of death among both cirrhotic patients with ARI and non-ARI control groups were liver-related (18.2% vs. 18.5%, *p* = 0.82) (Supplementary Table 3). The second most common cause of death also for both of these groups was related to cardiovascular disease, with a higher rate in the ARI group (5.1% vs. 3.6%, *p* = 0.023). As expected, the number of deaths caused by respiratory diseases in ARI patients was significantly higher than that among non-ARI patients (5.0% vs. 2.3%, *p* < 0.001). The results were similar for the 2010–11 season.

### Predictors for Mortality and Hospital Charges

#### Factors associated with risk of mortality

Multivariate Cox proportional hazard regression was performed to explore factors (age, sex, race, ethnicity, insurance type, severity of cirrhosis, cause of cirrhosis, comorbidities, and history of severe comorbidities) associated with mortality in cirrhotic patients in the 2011–12 influenza season, following PSM. The risk of dying during hospitalization was approximately 35% higher among cirrhotic patients with ARI than among cirrhotics without ARI (adjusted hazard ratio [aHR]) = 1.35 (95% CI: 1.22–1.49), *p* < 0.001). This trend was also consistent 30 days and 1 year after hospital discharge (30-day aHR = 1.13 (95% CI: 1.02–1.25), *p* = 0.023; 1-year aHR = 1.19 (95% CI: 1.07–1.32), *p* = 0.001). Hospitalized cirrhotic patients regardless of ARI status were more likely to die if they were older than 75 years of age compared to those younger than 45 years (in-hospital aHR = 2.94 (95% CI: 2.26–3.83), *p* < 0.001;30-day aHR = 1.85 (95% CI: 1.39–2.45), *p* < 0.001; 1-year aHR = 2.09 (95% CI: 1.58–2.76), *p* < 0.001). Cirrhotic patients with hepatocellular carcinoma (HCC) as well as decompensated liver disease also had a higher risk of mortality than patients with compensated cirrhosis. In addition, cirrhotic patients with alcoholic liver disease (ALD) had a 22% higher likelihood of dying during their hospitalization than those with cirrhosis from hepatitis B virus (HBV) infection (aHR = 1.22 (95% CI: 1.02–1.45), *p* = 0.028) (Table 2). Results from the 2010–11 season were similar (Supplementary Table 4).

**Table 2:** Multivariate analysis: factors associated with mortality in cirrhotic patients in the 2011–2012 season.

In-hospital/30-days/1-year number of death (n = 289/564/1486)	In-hospital mortality		Post-discharge mortality			
	Adjusted hazard ratio (95% CI)*	*P*-value	30 days		1 year	
			Adjusted hazard ratio (95% CI)*	*P-value*	Adjusted hazard ratio (95% CI)*	*P-value*
**ARI (n** = **100/192/471)**	**1.35 (1.22–1.49)**	<0.001	**1.13 (1.02–1.25)**	0.023	**1.19 (1.07–1.32)**	0.001
**Age**						
18–44 (n = 12/23/66)	Ref.		Ref.		Ref.	
45–64 (n = 148/283/774)	1.69 (1.35–2.12)	<0.001	1.19 (0.93–1.51)	0.17	1.20 (0.95–1.53)	0.13
65–74 (n = 66/134/362)	2.09 (1.63–2.68)	<0.001	1.50 (1.15–1.96)	0.003	1.61 (1.24–2.09)	<0.001
≥75 (n = 63/124/284)	2.94 (2.26–3.83)	<0.001	1.85 (1.39–2.45)	<0.001	2.09 (1.58–2.76)	<0.001
**Sex**						
Female (n = 95/190/548)	Ref.		Ref.		Ref.	
Male (n = 194/374/938)	1.03 (0.93–1.13)	0.59	1.05 (0.95–1.17)	0.36	1.04 (0.93–1.15)	0.51
**Race**						
White (n = 190/390/996)	Ref.		Ref.		Ref.	
Black (n = 26/48/141)	0.77 (0.66–0.91)	0.002	0.93 (0.78–1.11)	0.45	0.93 (0.78–1.11)	0.45
Native American/Alaskan/Aleutian (n = 2/2/10)	0.61 (0.32–1.15)	0.12	0.63 (0.31–1.28)	0.20	0.68 (0.34–1.38)	0.29
Asian/Pacific Islander (n = 36/55/143)	0.98 (0.81–1.18)	0.82	1.03 (0.84–1.26)	0.77	1.04 (0.86–1.28)	0.67
Others/unknown (n = 35/69/196)	0.92 (0.79–1.07)	0.27	0.98 (0.83–1.15)	0.79	0.97 (0.82–1.13)	0.67
**Ethnicity**						
Hispanic (n = 82/163/446)	Ref.		Ref.		Ref.	
Non-Hispanic (n = 204/396/1030)	0.92 (0.82–1.03)	0.14	0.89 (0.78–1.00)	0.058	0.85 (0.75–0.96)	0.01
Unknown (n = 3/5/10)	0.58 (0.34–0.99)	0.045	0.89 (0.50–1.58)	0.69	0.78 (0.44–1.39)	0. 40
**Insurance**						
Private (n = 28/63/159)	Ref.		Ref.		Ref.	
Medicare (n = 151/317/846)	0.90 (0.76–1.06)	0.20	0.95 (0.80–1.13)	0.55	0.89 (0.75–1.06)	0.18
Medicaid (n = 90/148/399)	0.85 (0.71–1.01)	0.065	0.91 (0.76–1.10)	0.33	0.88 (0.73–1.06)	0.18
Self-pay (n = 9/17/35)	0.90 (0.66–1.22)	0.50	0.89 (0.64–1.24)	0.49	0.86 (0.62–1.19)	0.36
Others^a^ (n = 11/19/47)	0.71 (0.53–0.94)	0.017	0.65 (0.48–0.88)	0.006	0.60 (0.44–0.82)	0.001
**Severity of cirrhosis**						
Compensated cirrhosis (n = 48/94/258)	Ref.		Ref.		Ref.	
Decompensated cirrhosis (n = 216/418/1113)	1.16 (1.02–1.31)	0.02	1.19 (1.05–1.36)	0.008	1.27 (1.11–1.44)	<0.001
Hepatocellular carcinoma (HCC) (n = 25/52/115)	2.17 (1.76–2.68)	<0.001	1.94 (1.55–2.42)	<0.001	2.35 (1.87–2.94)	<0.001
**Cause of cirrhosis**						
Hepatitis B virus^b^ (n = 33/59/179)	Ref.		Ref.		Ref.	
Hepatitis C virus^c^ (n = 122/259/741)	1.09 (0.94–1.26)	0.25	0.94 (0.81–1.09)	0.42	0.99 (0.85–1.15)	0.90
Alcoholic liver disease (ALD) (n = 88/168/361)	1.22 (1.02–1.45)	0.028	1.03 (0.85–1.23)	0.79	1.11 (0.92–1.33)	0.28
Non-alcoholic fatty liver disease (NAFLD) (n = 19/35/96)	0.95 (0.75–1.20)	0.65	0.95 (0.74–1.22)	0.70	1.01 (0.79–1.30)	0.91
Other liver diseases^d^ (n = 27/43/109)	0.80 (0.64–1.00)	0.051	0.95 (0.75–1.20)	0.65	0.95 (0.76–1.21)	0.70

*Adjusted for the listing variables, comorbidities, and history of severe comorbidities.

^a^Worker’s compensation, county indigent program, other government, other indigent program, other payer.

^b^HBV patients (with or without HCV, ALD, NAFLD).

^c^HCV patients (with or without ALD, NAFLD).

^d^Autoimmune hepatitis, alpha-1-antitrypsin deficiency, hereditary hemochromatosis, hemochromatosis due to repeated red blood cell transfusions, other hemochromatosis, disorders of copper metabolism, cholangitis.

#### Factors related to higher hospital charges

We adjusted for hospital days and other confounding factors while studying factors related to seasonal hospital charges for cirrhotic patients with ARI during the 2011–12 season (Supplementary Table 5). In cirrhotic patients with ARI, longer duration of hospitalization was significantly associated with increased seasonal hospital charges, and patients hospitalized for 3 to 15 days accumulated total hospital charges that on average were $66,194 higher than those among patients hospitalized for less than 2 days (*p* < 0.001). Cirrhotic patients with ARI and history of multi-organ failure accounted for an average of $30,132 in additional expenditures compared with ARI patients without history of multi-organ failure (*p* = 0.039).

## Discussion

We investigated the health burden of cirrhotic patients with and without ARI utilizing a large population-based sample of patients in the U.S, in which almost the entire inpatient population from the state of California was included. In addition to being the most populous state in the nation, with a total population of approximately 39 million, California’s population is also among the most ethnically and socioeconomically diverse. To our knowledge, this is the first study to investigate the financial and mortality burden associated with ARI in patients with cirrhosis. We found that the prevalence of ARI in hospitalized patients with cirrhosis ranged from 8% to 9% over the three consecutive influenza seasons investigated. The prevalence rate is significantly higher (almost 40-fold) than the prevalence rate of 0.22% for patients hospitalized with influenza as reported by CDC^15^.

The prevalence of ARI was also found to be significantly higher among older patients (>65), Asian and non-Hispanic patients, and those with Medicare or Medicaid insurance coverage. These findings are not surprising as acute respiratory illnesses strike the elderly in disproportionate numbers as well as those with chronic illnesses as often overly represented in these populations^16^.

We also found that the presence of ARI in patients with cirrhosis placed a tremendous burden on the health care system. Though, the total number of hospitalizations among cirrhotic patients with ARI was lower than that among cirrhotic patients without ARI, ARI patients had, on average, a significantly longer length of hospital stay, higher hospital charges per patient per admission, and higher hospital charges per patient per season than non-ARI cirrhotic patients. According to a 2011 study on cirrhotic patients requiring intensive care, the mean hospital charge for each admission was approximately $116,200^17^. This is close to the statistics in our study, where the average hospital charge per admission for cirrhotic patients with ARI in the 2011–12 season was $122,555.

Cirrhotic patients with ARI also experienced consistently higher mortality rates than cirrhotic inpatients without ARI. Compared with cirrhotic inpatients without ARI, cirrhotic patients with ARI had 35%, 13%, and 19% higher risk of in-hospital, 30-day and 1-year mortality, respectively. Furthermore, older age, HCC, and having ALD-related cirrhosis were each associated with higher risk of mortality.

Our results were also notable for a substantial risk of mortality among cirrhotic patients with ARI compared to the general population. In the general population, analysis of the NIS data previously reported a declining in-hospital mortality rate of 4.1% in 2005 from 8.9% in 1993 for community-acquired pneumonia^18^. In another study, in-hospital mortality of acute respiratory disease was also well below 10% at 5.0%, 4.3%, and 4.4% during the 2010–11, 2011–12, and 2012–13 influenza seasons respectively^19^. In contrast, the in-hospital mortality rate for cirrhotic patients with ARI in the current study was 7.6% during the 2011–2012 season, which was also higher than that among patients with cirrhosis but without ARI (5.8%).

Therefore, it is important to prevent ARI among cirrhotic patients. CDC reports on the effectiveness of the influenza vaccination for the years 2015–2016 estimated that the influenza vaccination prevented an estimated 5.1 million illnesses, 2.5 million medical visits, 71,000 hospitalizations, and 3,000 pneumonia and influenza deaths^15^. In addition, the CDC anticipated significant declines in invasive pneumococcal disease with the introduction of the pneumococcal conjugate vaccines in the United States (PCV7 in 2000 and PCV13 in 2010, 2012 for adults aged 19 years and older with immunocompromising disease and 2014 for adults >64 years of age), and estimates that invasive pneumococcal disease in adults 19 through 64 years old would decrease from 16 cases per 100,000 people in 1998 to only 7 cases per 100,000 people in 2015^20^. However, despite these impressive results, influenza vaccination coverage among adults aged ≥19 years was only 43.2% during the 2012–2013 influenza season while the pneumococcal vaccination coverage was only 20.3% among high-risk persons aged 19–64 years and 61.3% among adults aged ≥65 years during this same time^21^.

A significant strength of our study is that it is a population-based study utilizing a large and validated database and thus enhances its accuracy. We reported the updated prevalence, mortality and inpatient charge for cirrhotic patient overall and by specific etiologies to inform targeted public health measures for particularly high-risk groups such as older cirrhotic patients or those with alcoholic liver disease.

There are also several limitations to our study. This first is that the mortality rate from ARI might have been underestimated as a result of ARI diagnosis codes being miscoded or not recorded. Because the California Office of Statewide Health Planning and Development (OSHPD) database is an administrative database, the precision of diagnoses can also be decreased by additional coding errors. However, OSHPD has been a widely used and validated database and the accuracy of diagnoses is generally higher in inpatient than outpatient settings^22,23^. In addition, as the OSHPD database only contains data from patients in California, it may not represent the entire nation. However, California as the largest and most populous state in the U.S. and is worth studying in itself and with the most diverse population in the U.S., it also holds lessons that may be generalizable to multiple other regions in the country. Furthermore, California has a more temperate climate than many other states, which may have underestimated the overall impact of ARI and may have biased the outcomes towards the null hypothesis, and the impact of ARI is likely to be even greater in regions with more severe winter weather.

## Conclusions

Our findings suggest that ARI accounts for substantial morbidity, mortality and financial burden among hospitalized, cirrhotic patients in the U.S., especially among older, Medicare, Medicaid, Asian, and non-Hispanic patients, as well as those with history of HCC, ALD, or severe comorbidities. Cirrhotic patients, especially the higher risk groups, should be targeted for preventive measures such as influenza and pneumonia vaccination as well as early and effective outpatient management of those who have contracted ARI in order to prevent costly and high-risk hospital admissions.

## Methods

### Data Source

Data for this study were obtained from OSHPD and California State Death Statistical Master file records (DSMF). OSHPD is a longitudinal database that captures inpatient discharges from approximately 450 hospitals in California and 16 million inpatients. Patient records include demographics, insurance payer, facility type, and hospital charges. Primary diagnoses, principle procedures, and up to 24 secondary diagnoses and procedures are also reported in the OSHPD database^24^. Patients are each assigned a unique record linkage number (RLN), which is calculated based on social security numbers. Patients without social security numbers are classified as missing in RLN. The DSMF is a database of death certificates for all Californians. To form a complete record, DSMF is linked back to the OSHPD database through the RLNs.

This study was approved by the Institutional Review Board of the California Health and Human Services Agency in Sacramento, California and was considered exempt by the Stanford University Institutional Review Board, Stanford, California.

### Influenza Seasons

Three influenza seasons (2010–11, 2011–12, and 2012–13) were identified for this study. Influenza season dates were obtained from the California Department of Public Health Influenza Surveillance Program and were for 2010–2011 (October 3, 2010–May 21, 2011), for 2011–2012 (October 2, 2011–May 19, 2012), and for 2012–2013 (September 30, 2012–May 18, 2013)^25–27^.

### Definitions

Cirrhosis was determined by ICD 9 codes and clinically defined as the presence of clinical, radiologic, endoscopic, manifestations of cirrhosis and/or portal hypertension (thrombocytopenia, splenomegaly, presence of varices) or symptoms of clinical hepatic decompensation (ascites, hepatic encephalopathy, jaundice, variceal hemorrhage).

Acute respiratory illness was defined by influenza (ICD-9 code 487), pneumonia (ICD-9 code 480–486), and acute bronchitis/bronchiolitis (ICD-9 code 466) and included mechanical ventilation if relevant as noted by the ICD-9 procedure codes of 939, 967, 9670, 9671, 9672.

Severity of liver disease was categorized into compensated cirrhosis, decompensated cirrhosis, and HCC. Cause of cirrhosis covered the liver diseases of HBV, hepatitis C virus (HCV), ALD, non-alcoholic fatty liver disease, and other liver diseases.

### Sample Selection

All adult patients (≥18 years) diagnosed with cirrhosis (ICD-9 codes: Supplementary Table 6) in California before the start of each influenza season were included in the three influenza seasons from 2010–2013 detailed above.

We excluded patients with missing RLNs or co-infection with the human immunodeficiency virus. Patients without known cause of cirrhosis and who could not be categorized into one of the listed categories were excluded. We also excluded all patients who received a liver transplant at any time during our study period prior to any analysis.

#### Propensity Score Matching

We performed a 1:3 propensity score matching of cirrhotic patients with ARI vs. cirrhotic patients without ARI in each of the three influenza seasons to adjust for differences in background risks (age, sex, race, ethnicity, insurance type, hospital site, severity of cirrhosis, cause of cirrhosis, comorbidities, and history of severe comorbidities) between ARI cirrhotic patients and non-ARI cirrhotic controls. Nearest neighbour matching using the caliper of 0.009 was applied. To get the optimal result, 0.2 of standard deviation of the logit of propensity score was used to calculate the caliper as previously recommended^28^. We also examined the balance of measured covariates between the matched cohorts using standardized differences^29^.

### Outcomes

In patients with cirrhosis, we report the prevalence of ARI, number of hospitalizations, length of hospital stay, hospital charges per patient day, hospital charges per patient admission, hospital charges per patient season, and in-hospital as well as post-discharge mortality. Mortality analysis was not performed for the most recent influenza season (2012–2013) due to relative lack of follow-up data for this cohort. All charges were inflation (3%) adjusted to 2013 US dollars. Causes of death were identified by ICD 10 codes in OSHPD and we categorized the causes as related to liver disease (alcoholic cirrhosis of liver, HCV, other cirrhosis of liver, liver cancer, non-alcoholic liver disease), cardiovascular disease (coronary artery disease, heart failure, ST elevation myocardial infarction, stroke, etc.), respiratory disease (chronic obstructive pulmonary disease, pneumonia), other malignant neoplasms, diabetes mellitus and kidney disease.

Other comorbidities examined were obtained using ICD-9 codes and included cardiovascular disease, diabetes mellitus, hypertension, any cancer other than HCC, alcohol use/abuse, drug abuse, hyperlipidemia, chronic obstructive pulmonary disease, chronic kidney disease, mental illness, renal failure, cardiac failure, major neurologic events, severe hematologic conditions, multi-organ failure, sepsis, hepatic failure, and respiratory failure^16^.

The severity of liver disease, cause of cirrhosis, and all comorbidities were obtained prior to the beginning of each influenza season (ICD-9 codes: Supplementary Table 7).

### Statistical analysis

Chi-square test was used to perform the descriptive analysis on the prevalence of ARI since all demographic and clinical features were dichotomous. The multivariate generalized linear model (GLM) with a gamma distribution and log-link function was conducted to compare hospital charges. GLM with a Poisson distribution and log-link function was used to compare number of hospitalizations and length of hospital stay. We adjusted for age, sex, race, ethnicity, insurance type, severity of cirrhosis, cause of cirrhosis, comorbidities, and history of severe comorbidities. We also performed subgroup analysis to compare the seasonal hospital charge in patients who required mechanical ventilation and those who did not.

Kaplan-Meier survival analysis was used to describe the cumulative mortality rates of cirrhotic patients with and without ARI. The censor date was death or the end of follow up, whichever came first. We also performed a log rank test to examine the significance of the difference in mortality between ARI cases and non-ARI controls. The Cox proportional hazards regression model was used to estimate hazard ratios and its 95% confidence interval relating ARI with mortality for cirrhotic inpatients adjusted for age, sex, race, ethnicity, insurance type, severity of cirrhosis, cause of cirrhosis, comorbidities, and history of severe comorbidities.

All statistical analyses were 2-tailed, with a significance level of 0.05. We performed all analyses using STATA version 14 (Stata Corp., College Station, TX).

## Electronic supplementary material

Supplementary Information
