# Supplementary material for: Higher mortality and hospital charges in patients with cirrhosis and acute respiratory illness: a population-based study
Source: Sci Rep. 2018 Jul 2;8:9969. doi: 10.1038/s41598-018-28317-w (PMC6028654; doi:10.1038/s41598-018-28317-w)
Supplement: Supplementary file 1 — Supplementary Information [file 41598_2018_28317_MOESM1_ESM.docx]

**Title: Higher mortality and hospital charges in patients with cirrhosis and acute respiratory illness: a population-based study**

Biyao Zou, MPP^1^; Yee Hui Yeo, M.D., MSc^1^; Donghak Jeong, M.S. ^1^; Edward Sheen M.D., M.P.H., M.B.A. ^1^; Haesuk Park, Ph.D^2^; Pauline Nguyen, B.S.^1^; Yao-Chun Hsu, M.D, Ph.D^3^; Gabriel Garcia, M.D. ^1^; Mindie H. Nguyen, M.D. M.A.S. ^1^

Supplementary Table 1. Prevalence of acute respiratory illness (ARI) in cirrhotic patients and subgroups in the 2010-2011 and 2012-2013 seasons: overall cohort

| **Influenza seasons** | **2010-2011** | | | **2012-2013** | | |
| --- | --- | --- | --- | --- | --- | --- |
| Patient number  Covariates | **Cirrhotic patient (n= 14,049)** | | | **Cirrhotic patient (n= 16,444)** | | |
|  | **ARI = 1240 (%)** | **Non-ARI = 12,809 (%)** | ***P*-value** | **ARI = 1410 (%)** | **Non-ARI = 15,034 (%)** | ***P*-value** |
| **Age** |  | | | | | |
| 18-44 | 74 (4.9) | 1442 (95.1) | <.001 | 72 (4.5) | 1541 (95.5) | <.001 |
| 45-64 | 783 (8.7) | 8213 (91.3) |  | 790 (7.6) | 9591 (92.4) |  |
| 65-74 | 215 (10.0) | 1939 (90.0) |  | 320 (11.3) | 2500 (88.7) |  |
| ≥75 | 168 (12.1) | 1215 (87.9) |  | 228 (14.0) | 1402 (86.0) |  |
| **Sex** |  | | | | | |
| Female | 465 (8.7) | 4894 (91.3) | 0.62 | 525 (8.5) | 5681 (91.5) | 0.68 |
| Male | 775 (8.9) | 7915 (91.1) |  | 885 (10.3) | 9353 (89.7) |  |
| **Race** |  | | | | | |
| White | 853 (8.7) | 8914 (91.3) | 0.42 | 946 (8.3) | 10,477 (91.7) | 0.014 |
| Black | 124 (9.2) | 1226 (90.8) |  | 158 (10.3) | 1374 (89.7) |  |
| Native American/Alaskan/Aleutian | 10 (8.7) | 105 (91.3) |  | 8 (5.2) | 146 (94.8) |  |
| Asian/Pacific Islander | 91 (10.5) | 777 (89.5) |  | 108 (10.1) | 964 (89.9) |  |
| Others/unknown | 162 (8.3) | 1787 (91.7) |  | 190 (8.4) | 2073 (91.6) |  |
| **Ethnicity** |  | | | | | |
| Hispanic | 357 (7.8) | 4224 (92.2) | 0.01 | 399 (7.6) | 4843 (92.4) | 0.01 |
| Non-Hispanic | 874 (9.3) | 8509 (90.7) |  | 1000 (9.0) | 10,075 (91.0) |  |
| Unknown | 9 (10.6) | 76 (89.4) |  | 11 (8.7) | 116 (91.3) |  |
| **Insurance** |  | | | | | |
| Medicare | 603 (10.1) | 5339 (89.9) | <.001 | 773 (10.7) | 6429 (89.3) | <.001 |
| Medicaid | 418 (9.2) | 4108 (90.8) |  | 415 (8.4) | 4545 (91.6) |  |
| Private | 127 (7.0) | 1685 (93.0) |  | 113 (5.4) | 1974 (94.6) |  |
| Self-pay | 34 (4.5) | 726 (95.5) |  | 35 (4.9) | 674 (95.1) |  |
| Others^a^ | 58 (5.7) | 951 (94.3) |  | 74 (5.0) | 1412 (95.0) |  |
| **Severity of cirrhosis** |  | | | | | |
| Compensated cirrhosis | 263 (9.2) | 2606 (90.8) | 0.03 | 237 (8.1) | 2672 (91.9) | 0.55 |
| Decompensated cirrhosis | 935 (8.9) | 9554 (91.1) |  | 1108 (8.7) | 11,624 (91.3) |  |
| Hepatocellular carcinoma (HCC) | 42 (6.1) | 649 (93.9) |  | 65 (8.1) | 738 (91.9) |  |
| **Cause of cirrhosis** |  | | | | | |
| Hepatitis B virus^b^ | 134 (8.2) | 1492 (91.8) | 0.025 | 188 (10.3) | 1638 (89.7) | <.001 |
| Hepatitis C virus^c^ | 686 (9.6) | 6469 (90.4) |  | 730 (8.9) | 7506 (91.1) |  |
| Alcoholic liver disease (ALD) | 283 (7.9) | 3317 (92.1) |  | 300 (7.0) | 3966 (93.0) |  |
| Non-alcoholic fatty liver disease (NAFLD) | 58 (7.7) | 692 (92.3) |  | 99 (10.0) | 887 (90.0) |  |
| Other liver diseases^d^ | 79 (8.6) | 839 (91.4) |  | 93 (8.2) | 1037 (91.8) |  |

*adjusted for comorbidities and history of severe comorbidities

a: worker’s compensation, county indigent program, other government, other indigent program, other payer

b: HBV patients (with or without HCV, ALD, NAFLD)

c: HCV patients (with or without ALD, NAFLD)

d: Autoimmune hepatitis, alpha-1-antitrypsin deficiency, hereditary hemochromatosis, hemochromatosis due to repeated red blood cell transfusions, other hemochromatosis, disorders of copper metabolism, cholangitis

Supplementary Table 2. Characteristics of cirrhotic patients with and without acute respiratory illness (ARI) in the 2010-2011 and 2012-2013 seasons: after propensity score matching

| **Influenza seasons** | **2010-2011** | | |  | **2012-2013** | | |  |
| --- | --- | --- | --- | --- | --- | --- | --- | --- |
| **Patient number** | **Cirrhotic patient (n= 4244)** | | |  | **Cirrhotic patient (n= 4791)** | | |  |
|  | **ARI** | **No ARI** | ***P*-value** | **Standardized difference** | **ARI** | **No ARI** | ***P*-value** | **Standardized difference** |
| **Covariates** | n = 1240 (%) | n = 3004 (%) |  |  | n = 1409 (%) | n = 3382 (%) |  |  |
| **Age** |  |  |  |  |  |  |  |  |
| 18-44 | 74 (6.0) | 243 (8.1) | 0.86 | 0.7 | 72 (5.1) | 181 (5.4) | 0.80 | -1.0 |
| 45-64 | 783 (63.2) | 1842 (61.3) |  |  | 790 (56.1) | 1983 (58.6) |  |  |
| 65-74 | 215 (17.3) | 534 (17.8) |  |  | 320 (22.7) | 715 (21.1) |  |  |
| ≥75 | 168 (13.6) | 385 (12.8) |  |  | 227 (16.1) | 503 (14.9) |  |  |
| **Sex** |  |  |  |  |  |  |  |  |
| Female | 465 (37.5) | 1062 (35.4) | 0.12 | -6.2 | 524 (37.2) | 1296 (38.3) | 0.67 | 1.6 |
| Male | 775 (62.5) | 1942 (64.7) |  |  | 885 (62.8) | 2086 (61.7) |  |  |
| **Race** |  |  |  |  |  |  |  |  |
| White | 853 (68.8) | 2080 (69.2) | 0.92 | 0.4 | 946 (67.1) | 2299 (68.0) | 0.75 | 1.2 |
| Black | 124 (10.0) | 307 (10.2) |  |  | 158 (11.2) | 340 (10.1) |  |  |
| Native American/Alaskan/Aleutian | 10 (0.81) | 19 (0.63) |  |  | 8 (0.57) | 39 (1.2) |  |  |
| Asian/Pacific Islander | 91 (7.3) | 201 (6.7) |  |  | 108 (7.7) | 274 (8.1) |  |  |
| Others/unknown | 162 (13.1) | 397 (13.2) |  |  | 189 (13.4) | 430 (12.7) |  |  |
| **Ethnicity** |  |  |  |  |  |  |  |  |
| Hispanic | 357 (28.8) | 880 (29.3) | 0.89 | -0.5 | 399 (28.3) | 962 (28.4) | 0.56 | -2.2 |
| Non-Hispanic | 874 (70.5) | 2107 (70.1) |  |  | 999 (70.9) | 2397 (70.9) |  |  |
| Unknown | 9 (0.73) | 17 (0.57) |  |  | 11 (0.78) | 23 (0.68) |  |  |
| **Insurance** |  |  |  |  |  |  |  |  |
| Medicare | 603 (48.6) | 1495 (50.0) | 0.80 | -1.0 | 772 (54.8) | 1815 (53.7) | 0.82 | 0.8 |
| Medicaid | 418 (33.7) | 925 (30.8) |  |  | 415 (29.5) | 948 (28.0) |  |  |
| Private | 127 (10.2) | 300 (10.0) |  |  | 113 (8.0) | 331 (9.8) |  |  |
| Self-pay | 34 (2.7) | 123 (4.1) |  |  | 35 (2.5) | 92 (2.7) |  |  |
| Others^a^ | 58 (4.7) | 161 (5.4) |  |  | 74 (5.3) | 196 (5.8) |  |  |
| **Severity of Cirrhosis** |  |  |  |  |  |  |  |  |
| Compensated cirrhosis | 263 (21.2) | 669 (22.3) | 0.93 | -0.4 | 237 (16.8) | 585 (17.3) | 0.78 | -1.0 |
| Decompensated cirrhosis | 935 (75.4) | 2218 (72.6) |  |  | 1107 (78.6) | 2615 (77.3) |  |  |
| Hepatocellular carcinoma (HCC) | 42 (3.4) | 153 (5.1) |  |  | 65 (4.6) | 182 (5.4) |  |  |
| **Cause of Cirrhosis** |  |  |  |  |  |  |  |  |
| Hepatitis B virus^b^ | 134 (10.8) | 381 (12.7) | 0.91 | 0.4 | 187 (13.3) | 436 (12.9) | 0.77 | -1.1 |
| Hepatitis C virus^c^ | 686 (55.3) | 1562 (52.0) |  |  | 730 (51.8) | 1704 (50.4) |  |  |
| Alcoholic liver disease (ALD) | 283 (22.8) | 706 (23.5) |  |  | 300 (21.3) | 790 (23.4) |  |  |
| Non-alcoholic fatty liver disease (NAFLD) | 58 (4.7) | 160 (5.3) |  |  | 99 (7.0) | 218 (6.5) |  |  |
| Other liver diseases^d^ | 79 (6.4) | 195 (6.5) |  |  | 93 (6.6) | 234 (6.9) |  |  |
| **Comorbidities** |  |  |  |  |  |  |  |  |
| Cardiovascular disease | 621 (50.1) | 1399 (46.6) | 0.55 | 2.5 | 811 (57.6) | 1875 (55.4) | 0.73 | -1.3 |
| Diabetes mellitus | 484 (39.0) | 1156 (38.5) | 0.90 | -0.5 | 614 (43.6) | 1451 (42.9) | 0.92 | 0.4 |
| Hypertension | 790 (63.7) | 1891 (63.0) | 0.99 | -0.1 | 964 (68.4) | 2287 (67.6) | 0.89 | -0.5 |
| Any cancer (other than HCC) | 87 (7.0) | 224 (7.5) | 0.43 | -3.4 | 132 (9.4) | 297 (8.8) | 0.94 | -0.3 |
| Alcohol use/abuse | 663 (53.5) | 1637 (54.5) | 0.86 | -0.7 | 734 (52.1) | 1792 (53.0) | 0.73 | 1.3 |
| Drug abuse | 692 (55.8) | 1631 (54.3) | 0.90 | 0.5 | 764 (54.2) | 1795 (53.1) | 0.43 | 3.0 |
| Hyperlipidemia | 274 (22.1) | 664 (22.1) | 1.00 | 0.0 | 456 (32.4) | 1061 (31.4) | 0.94 | -0.3 |
| Chronic obstructive pulmonary disease | 533 (43.0) | 1128 (37.6) | 0.58 | 2.4 | 601 (42.7) | 1333 (39.4) | 0.98 | -0.1 |
| Chronic kidney disease | 199 (16.1) | 497 (16.5) | 0.29 | -4.6 | 284 (20.2) | 642 (19.0) | 0.94 | 0.3 |
| Mental illness | 210 (16.9) | 493 (16.4) | 0.77 | 1.1 | 248 (17.6) | 580 (17.2) | 0.82 | 0.9 |
| **History of severe comorbidities** |  |  |  |  |  |  |  |  |
| Renal failure | 388 (31.3) | 937 (31.2) | 0.59 | -2.2 | 581 (41.2) | 1375 (40.7) | 0.77 | -1.1 |
| Cardiac failure | 367 (29.6) | 837 (27.9) | 0.62 | -2.1 | 483 (34.3) | 1085 (32.1) | 0.93 | 0.4 |
| Major neurologic events | 122 (9.8) | 291 (9.7) | 0.86 | -0.7 | 163 (11.6) | 345 (10.2) | 0.21 | 4.8 |
| Severe hematologic conditions | 301 (24.3) | 716 (23.8) | 0.83 | 0.9 | 368 (26.1) | 864 (25.6) | 0.88 | 0.6 |
| Multi-organ failure | 142 (11.5) | 309 (10.3) | 0.99 | -0.1 | 228 (16.2) | 483 (14.3) | 0.70 | 1.6 |
| Sepsis | 248 (20.0) | 560 (18.6) | 0.96 | 0.2 | 345 (24.5) | 802 (23.7) | 0.64 | -1.9 |
| Hepatic failure | 151 (12.2) | 352 (11.7) | 0.37 | 3.5 | 176 (12.5) | 442 (13.1) | 0.59 | -2.0 |
| Respiratory failure | 267 (21.5) | 587 (19.5) | 0.75 | -1.4 | 358 (25.4) | 744 (22.0) | 0.48 | 2.9 |

a: worker’s compensation, county indigent program, other government, other indigent program, other payer

b: HBV patients (with or without HCV, ALD, NAFLD)

c: HCV patients (with or without ALD, NAFLD)

d: Autoimmune hepatitis, alpha-1-antitrypsin deficiency, hereditary hemochromatosis, hemochromatosis due to repeated red blood cell transfusions, other hemochromatosis, disorders of copper metabolism, cholangitis

Supplementary Table 3. Causes of death for cirrhotic patients with and without acute respiratory illness (ARI) in the 2011-2012 season

Supplementary Table 4. Multivariate analysis: factors associated with mortality in cirrhotic patients in the 2010-2011 season

| **In-hospital/30-days/1-year number of death**  **(n= 296/581/1453)** | **In-hospital mortality** | | **Post-discharge mortality** | | | |
| --- | --- | --- | --- | --- | --- | --- |
|  | **Adjusted hazard ratio (95% CI)** | ***P*-value** | **30 days** | | **1 year** | |
|  |  |  | **Adjusted hazard ratio (95% CI)** | ***P-value*** | **Adjusted hazard ratio (95% CI)** | ***P-value*** |
| **ARI (n= 117/214/484)** | **1.39 (1.27-1.52)** | <.001 | **1.09 (0.99-1.20)** | 0.098 | **1.17 (1.07-1.29)** | 0.001 |
| **Age** |  |  |  |  |  |  |
| 18-44 (n=9/18/54) | Ref. |  | Ref. |  | Ref. |  |
| 45-64 (n=172/330/822) | 1.71 (1.38-2.12) | <.001 | 1.48 (1.18-1.84) | 0.001 | 1.58 (1.27-1.97) | <.001 |
| 65-74 (n=60/120/308) | 2.11 (1.66-2.68) | <.001 | 1.94 (1.52-2.48) | <.001 | 2.39 (1.87-3.06) | <.001 |
| ≥75 (n=55/113/269) | 2.19 (1.70-2.83) | <.001 | 2.17 (1.67-2.81) | <.001 | 2.71 (2.09-3.51) | <.001 |
| **Sex** |  |  |  |  |  |  |
| Female (n=103/200/504) | Ref. |  | Ref. |  | Ref. |  |
| Male (n=193/381/949) | 0.87 (0.80-0.96) | 0.005 | 1.07 (0.97-1.19) | 0.15 | 1.09 (0.99-1.21) | 0.071 |
| **Race** |  |  |  |  |  |  |
| White (n=196/395/996) | Ref. |  | Ref. |  | Ref. |  |
| Black (n=24/48/130) | 0.97 (0.83-1.13) | 0.69 | 0.96 (0.82-1.13) | 0.65 | 0.96 (0.82-1.12) | 0.58 |
| Native American/Alaskan/Aleutian (n=1/1/12) | 0.84 (0.51-1.37) | 0.48 | 0.92 (0.56-1.51) | 0.74 | 0.91 (0.55-1.51) | 0.72 |
| Asian/Pacific Islander (n=32/55/120) | 1.14 (0.96-1.36) | 0.15 | 0.94 (0.78-1.13) | 0.49 | 0.98 (0.81-1.18) | 0.81 |
| Others/unknown (n=43/82/195) | 1.01 (0.87-1.16) | 0.91 | 0.92 (0.79-1.07) | 0.26 | 0.90 (0.78-1.05) | 0.19 |
| **Ethnicity** |  |  |  |  |  |  |
| Hispanic (n=87/163/424) | Ref. |  | Ref. |  | Ref. |  |
| Non-Hispanic (n=203/410/1017) | 0.93 (0.84-1.04) | 0.22 | 0.98 (0.87-1.10) | 0.68 | 0.98 (0.88-1.10) | 0.79 |
| Unknown (n=6/8/12) | 0.97 (0.59-1.59) | 0.90 | 1.06 (0.58-1.95) | 0.84 | 1.19 (0.65-2.19) | 0. 57 |
| **Insurance** |  |  |  |  |  |  |
| Private (n=36/72/152) | Ref. |  | Ref. |  | Ref. |  |
| Medicare (n=153/308/782) | 0.90 (0.77-1.05) | 0.19 | 0.94 (0.79-1.10) | 0.43 | 0.90 (0.77-1.07) | 0.23 |
| Medicaid (n=91/171/442) | 0.71 (0.61-0.83) | <.001 | 0.95 (0.80-1.12) | 0.53 | 0.98 (0.83-1.15) | 0.78 |
| Self-pay (n=9/15/34) | 0.74 (0.54-1.01) | 0.058 | 0.71 (0.51-0.99) | 0.041 | 0.70 (0.50-0.97) | 0.033 |
| Others^a^ (n=7/15/43) | 0.70 (0.53-0.91) | 0.009 | 0.73 (0.55-0.96) | 0.025 | 0.69 (0.52-0.91) | 0.008 |
| **Severity of cirrhosis** |  |  |  |  |  |  |
| Compensated cirrhosis (n=47/99/261) | Ref. |  | Ref. |  | Ref. |  |
| Decompensated cirrhosis (n=225/423/1079) | 0.98 (0.88-1.10) | 0.78 | 1.04 (0.93-1.17) | 0.46 | 1.10 (0.98-1.23) | 0.11 |
| Hepatocellular carcinoma (HCC) (n=24/59/113) | 1.40 (1.15-1.70) | 0.001 | 1.68 (1.37-2.06) | <.001 | 2.10 (1.71-2.58) | <.001 |
| **Cause of cirrhosis** |  |  |  |  |  |  |
| Hepatitis B virus^b^ (n=30/57/151) | Ref. |  | Ref. |  | Ref. |  |
| Hepatitis C virus^c^ (n=147/301/705) | 0.98 (0.85-1.13) | 0.78 | 1.02 (0.88-1.18) | 0.82 | 1.03 (0.89-1.19) | 0.66 |
| Alcoholic liver disease (ALD) (n=92/161/389) | 1.04 (0.88-1.22) | 0.67 | 1.03 (0.87-1.22) | 0.73 | 1.09 (0.92-1.29) | 0.34 |
| Non-alcoholic fatty liver disease (NAFLD) (n=12/26/92) | 1.11 (0.89-1.40) | 0.35 | 1.24 (0.99-1.56) | 0.067 | 1.47 (1.17-1.85) | 0.001 |
| Other liver diseases^d^ (n=15/36/116) | 1.12 (0.91-1.38) | 0.29 | 1.17 (0.94-1.44) | 0.16 | 1.34 (1.08-1.65) | 0.007 |

*adjusted for comorbidities and history of severe comorbidities

a: worker’s compensation, county indigent program, other government, other indigent program, other payer

b: HBV patients (with or without HCV, ALD, NAFLD)

c: HCV patients (with or without ALD, NAFLD)

d: Autoimmune hepatitis, alpha-1-antitrypsin deficiency, hereditary hemochromatosis, hemochromatosis due to repeated red blood cell transfusions, other hemochromatosis, disorders of copper metabolism, cholangitis

Supplementary Table 5. Multivariate analysis: factors associated with seasonal hospital charge for cirrhotic patients with acute respiratory illness (ARI) in the 2011-2012 season

| **Number of ARI patients (n = 1311)** | **P. mean difference in charge** | ***P-value*** |
| --- | --- | --- |
| **Age** |  |  |
| 18-44 (n = 75) | Ref. |  |
| 45-64 (n = 774) | -24,485 | 0.22 |
| 65-74 (n = 290) | -17,529 | 0.42 |
| ≥75 (n = 172) | -35,071 | 0.14 |
| **Sex** |  |  |
| Female (n = 498) | Ref. |  |
| Male (n = 813) | -146 | 0.99 |
| **Race** |  |  |
| White (n = 894) | Ref. |  |
| Black (n = 145) | 1053 | 0.94 |
| Native American/Alaskan/Aleutian (n = 10) | -13,297 | 0.76 |
| Asian/Pacific Islander (n = 107) | 5097 | 0.77 |
| Others/unknown (n = 155) | 11,043 | 0.44 |
| **Ethnicity** |  |  |
| Hispanic (n = 366) | Ref. |  |
| Non-Hispanic (n = 941) | 18,080 | 0.074 |
| Unknown (n = 4) | 85,144 | 0.39 |
| **Insurance** |  |  |
| Private (n = 132) | Ref. |  |
| Medicare (n = 669) | -11,456 | 0.45 |
| Medicaid (n = 407) | -4460 | 0.77 |
| Self-pay (n = 42) | -45,204 | 0.051 |
| Others^a^ (n = 61) | -28,410 | 0.19 |
| **Days in hospital** |  |  |
| ≤ 2 (n = 49) | Ref. |  |
| 3-15 (n = 791) | 66,194 | <.001 |
| 16-45 (n = 385) | 292,233 | <.001 |
| ≥ 46(n = 86) | 791,032 | <.001 |
| **Severity of cirrhosis** |  |  |
| Compensated cirrhosis (n = 283) | Ref. |  |
| Decompensated cirrhosis (n = 975) | 9738 | 0.33 |
| Hepatocellular carcinoma (HCC) (n = 53) | -32,956 | 0.081 |
| **Cause of cirrhosis** |  |  |
| Hepatitis B virus^b^ (n = 155) | Ref. |  |
| Hepatitis C virus^c^ (n = 712) | 3139 | 0.81 |
| Alcoholic liver disease (ALD) (n = 285) | 5455 | 0.72 |
| Non-alcoholic fatty liver disease (NAFLD) (n = 82) | 18,448 | 0.39 |
| Other liver diseases^d^ (n = 77) | 39,372 | 0.083 |
| **Comorbidities** |  |  |
| Cardiovascular disease (n = 715) | -8313 | 0.39 |
| Diabetes mellitus (n = 537) | 8585 | 0.35 |
| Hypertension (n = 876) | 1586 | 0.86 |
| Any cancer (other than HCC) (n = 107) | 4412 | 0.77 |
| Alcohol use/abuse (n = 669) | 6565 | 0.50 |
| Drug abuse (n = 707) | 13,050 | 0.18 |
| Hyperlipidemia (n = 341) | -16,002 | 0.11 |
| Chronic obstructive pulmonary disease (n = 539) | 13,493 | 0.15 |
| Chronic kidney disease (n = 233) | 17,613 | 0.19 |
| Mental illness (n = 208) | -38,199 | <.001 |
| **History of severe comorbidities** |  |  |
| Renal failure (n = 498) | 1174 | 0.91 |
| Cardiac failure (n = 413) | 2483 | 0.81 |
| Major neurologic events (n = 123) | 18,479 | 0.23 |
| Severe hematologic conditions (n = 343) | 4203 | 0.66 |
| Multi-organ failure (n = 188) | 30,132 | 0.039 |
| Sepsis (n = 304) | -8127 | 0.46 |
| Hepatic failure (n = 164) | -21,112 | 0.073 |
| Respiratory failure (n=303) | 9751 | 0.39 |

a: worker’s compensation, county indigent program, other government, other indigent program, other payer

b: HBV patients (with or without HCV, ALD, NAFLD)

c: HCV patients (with or without ALD, NAFLD)

d: Autoimmune hepatitis, alpha-1-antitrypsin deficiency, hereditary hemochromatosis, hemochromatosis due to repeated red blood cell transfusions, other hemochromatosis, disorders of copper metabolism, cholangitis

Supplementary Table 6. ICD 9 codes for liver cirrhosis

| **Liver cirrhosis** |  |  | **condition: primary liver disease** |
| --- | --- | --- | --- |
| **Severity 1. compensated cirrhosis** |  |  |  |
| Alcoholic cirrhosis of liver | ICD-9 Dx | 571.2 | unconditioned |
| Cirrhosis of liver without mention of alcohol | ICD-9 Dx | 571.5 | unconditioned |
| Biliary Cirrhosis | ICD-9 Dx | 571.6 | unconditioned |
| Portal hypertension | ICD-9 Dx | 572.3 | condition |
| Splenomegaly | ICD-9 Dx | 789.2 | condition |
| **Severity 2. Decompensated cirrhosis** |  |  |  |
| Esophageal varices with bleeding | ICD-9 Dx | 456 | condition |
| Esophageal varices without mention of bleeding | ICD-9 Dx | 456.1 | condition |
| Esophageal varices in diseases classified elsewhere | ICD-9 Dx | 456.2 | condition |
| Esophageal varices in diseases classified elsewhere, with bleeding | ICD-9 Dx | 456.20 | condition |
| Esophageal varices in diseases classified elsewhere, without mention of bleeding | ICD-9 Dx | 456.21 | condition |
| Vulval varices | ICD-9 Dx | 456.6 | condition |
| Unspecified hemorrhoids with other complication | ICD-9 Dx | 455.8 | condition |
| Encephalopathy, not elsewhere classified | ICD-9 Dx | 348.3% | condition |
| Other ascites | ICD-9 Dx | 789.59 | condition |
| Hepatic encephalopathy | ICD-9 Dx | 572.2 | condition |
| Hepatorenal syndrome | ICD-9 Dx | 572.4 | unconditioned |
| Jaundice, unspecified, not of newborn | ICD-9 Dx | 782.4 | condition |
| Esophageal hemorrhage | ICD-9 Dx | 530.82 | condition |
| Gastrointestinal hemorrhage or esophageal varices (456.x) | ICD-9 Dx | 578.% | condition |
| Spontaneous bacterial peritonitis | ICD-9 Dx | 567.23 | unconditioned |
| Hyponatremia | ICD-9 Dx | 276.1 | condition |
| Hypoalbuminemia | ICD-9 Dx | 273.8 | condition |
| Acquired coagulation factor deficiency | ICD-9 Dx | 286.7 | condition |
| Other and unspecified coagulation defects | ICD-9 Dx | 286.9 | condition |
| Hepatic hydrothorax (pleural effusion with ascites) | ICD-9 Dx | 511.8, 511.9 and 789.59 | condition |
| Paracentesis | CPT, ICD-9 proc codes | 49080, 49081, 49082, 49083, 54.91 | condition |
| Esophagoscopy, rigid or flexible, with injection sclerosis of esophageal varices, Endoscopic excision or destruction of lesion or tissue of esophagus | CPT, ICD-9 proc codes | 43204, 42.33 | condition |
| Ligation of esophageal varices | ICD-9 proc codes | 42.91 | condition |
| Ligation of gastric varices | ICD-9 proc codes | 44.91 | condition |
| Insertion of Sengstaken tube | ICD-9 proc codes | 96.06 | condition |
| TIPS (Intra-abdominal venous shunt, Insertion of transvenous intrahepatic portosystemic shunt, Revision of transvenous intrahepatic portosystemic shunt) | CPT, ICD-9 proc codes | 37182, 37183, 39.1 | condition |
| venous anastomosis, open; portocaval | CPT codes | 37140 | condition |
| venous anastomosis, open; caval-mesenteric | CPT codes | 37160 | condition |
| venous anastomosis, open; splenorenal, proximal | CPT codes | 37180 | condition |
| venous anastomosis, open; splenorenal, distal (selective decompression of esophagogastric varices, any technique) | CPT codes | 37181 | condition |
| esophagoscopy, rigid or flexible; with band ligation of esophageal varices | CPT codes | 43205 | condition |
| upper gastrointestinal endoscopy including esophagus, stomach, and either the duodenum and/or jejunum as appropriate; with injection sclerosis | CPT codes | 43243 | condition |
| upper gastrointestinal endoscopy including esophagus, stomach, and either the duodenum and/or jejunum as appropriate; with band ligation of eso | CPT codes | 43244 | condition |
| ligation, direct, esophageal varices | CPT codes | 43400 | condition |
| transection of esophagus with repair, for esophageal varices | CPT codes | 43401 | condition |
| **Severity 3. Hepatocellular carcinoma** |  |  |  |
| Malignant neoplasm of liver and intrahepatic bile ducts | ICD-9 Dx | 155.% | unconditioned |

Supplementary Table 7. ICD-9 codes for comorbidity

|  | **ICD-9 CM & Code Description** |
| --- | --- |
| Cardiovascular disease | 390.x-392.x Acute rheumatic fever  393.x-398.x Chronic rheumatic heart disease  410.x-414.x ischemic heart disease  415.x-416.x acute pulmonary heart disease and chronic pulmonary heart disease  420.x-429.x Other forms of heart disease (exclude 428X)  440.x atherosclerosis |
| Diabetes mellitus | 250.% |
| Hypertension | 403.00, 403.10, 403.90, 404.00, 404.01, 404.30, 404.31, 404.90, 404.91, 401.x  402.x hypertensive heart disease |
| Any cancer (other than HCC) | 140 – 172.99, 174-195.99, 200-208.99 |
| Alcohol dependence/abuse | alcohol-induced mental disorder (291.x),  alcohol dependence syndrome (303.x),  alcohol abuse (305.0x),  personal history of mental disorder: alcoholism (V11.3),  alcoholic polyneuropathy (357.5),  alcoholic cardiomyopathy (425.5),  alcoholic gastritis (535.3x) |
| Injection/non-injection drug abuse | drug psychosis (292.x),  drug dependence (304.x),  tobacco use disorder (305.1x),  cannabis abuse (305.2x),  hallucinogen abuse (305.3x),  barbiturate abuse (305.4x),  opioid abuse (305.5x),  cocaine abuse (305.6x),  amphetamine abuse (305.7x),  antidepressant type abuse (305.8x),  other, mixed or unspecified drug abuse (305.9x) |
| Hyperlipidemia | 272.0, 272.1, 272.2, 272.4 |
| COPD | 490-492 (emphysema)  494 Bronchiectasis  496 Chronic airway obstruction, not elsewhere classified |
| Chronic Kidney disease and dialysis | CKD stage 3 (585.3),  CKD stage 4 (585.4),  CKD stage 5 (585.5),  ESRD (585.6),  glomerulonephritis (582x),  proteinuria (791.0),  nephrotic syndrome (581) |
| Serious mental illness | Bipolar disorder (296.0x, 296.4x,296.5x, 296.6x, 296.7x, 296.8x),  major depressive disorder (296.2-296.3),  schizophrenia (295.xx) |
| **History of severe comorbidities** |  |
| Cardiac failure | 428X  785.51 cardiogenic shock |
| Renal Failure | 586X Renal failure, unspecified  584X acute kidney failure  585.6 end-stage renal disease |
| major neurologic events | 348.3 encephalopathy, not elsewhere classified  293X transient mental disorders due to conditions classified elsewhere  348.1 anoxic brain damage  780.01 coma  345.1X, 345.3X, 345.4X, 345.51, 345.71, 345.81, 345.91 epilepsy and recurrent seizures (seizure, status epilepticus)  435X transient cerebral ischemia (transient ischemic accident)  cerebrovascular accident (embolic, hemorrhage)  430X, 431X, 432X, 433X, 434X, 436X, 437X |
| severe hematologic conditions | 286.6 Defibrination syndrome  41.0 Bone marrow or hematopoietic stem cell transplant  284X Aplastic anemia and other bone marrow failure syndromes  [200](http://www.icd9data.com/2012/Volume1/140-239/200-209/200/default.htm)X Lymphosarcoma and reticulosarcoma and other specified malignant tumors of lymphatic tissue  [201](http://www.icd9data.com/2012/Volume1/140-239/200-209/201/default.htm)X Hodgkin's disease  [202](http://www.icd9data.com/2012/Volume1/140-239/200-209/202/default.htm)X Other malignant neoplasms of lymphoid and histiocytic tissue  [203](http://www.icd9data.com/2012/Volume1/140-239/200-209/203/default.htm)X Multiple myeloma and immunoproliferative neoplasms  [204](http://www.icd9data.com/2012/Volume1/140-239/200-209/204/default.htm)X Lymphoid leukemia  [205](http://www.icd9data.com/2012/Volume1/140-239/200-209/205/default.htm)X Myeloid leukemia  [206](http://www.icd9data.com/2012/Volume1/140-239/200-209/206/default.htm)X Monocytic leukemia  [207](http://www.icd9data.com/2012/Volume1/140-239/200-209/207/default.htm)X Other specified leukemia  [208](http://www.icd9data.com/2012/Volume1/140-239/200-209/208/default.htm)X Leukemia of unspecified cell type  [453](http://www.icd9data.com/2013/Volume1/390-459/451-459/453/default.htm)X Other venous embolism and thrombosis |
| multi-organ failure | 995.92 Systemic inflammatory response syndrome due to infectious process with organ dysfunction (multi-organ failure) |
| Sepsis | 995.90 Systemic inflammatory response syndrome, unspecified (SIRS)  995.91 Sepsis  785.52 Septic shock |
| Hepatic failure | 570X Acute and subacute necrosis of liver (fulminant hepatic failure)  573.4 Hepatic infarction  572.8 end stage liver disease |
| Respiratory failure | 518.81 acute respiratory failure  518.82 ARDS  518.83 chronic respiratory failure |

Supplementary Figure 1. Adjusted hospital charges per patient per season, per admission, per day, number of hospitalizations per season and length of hospital stay per season for cirrhotic patients with and without acute respiratory illness (ARI) in 2010-11, 2011-12 and 2012-13


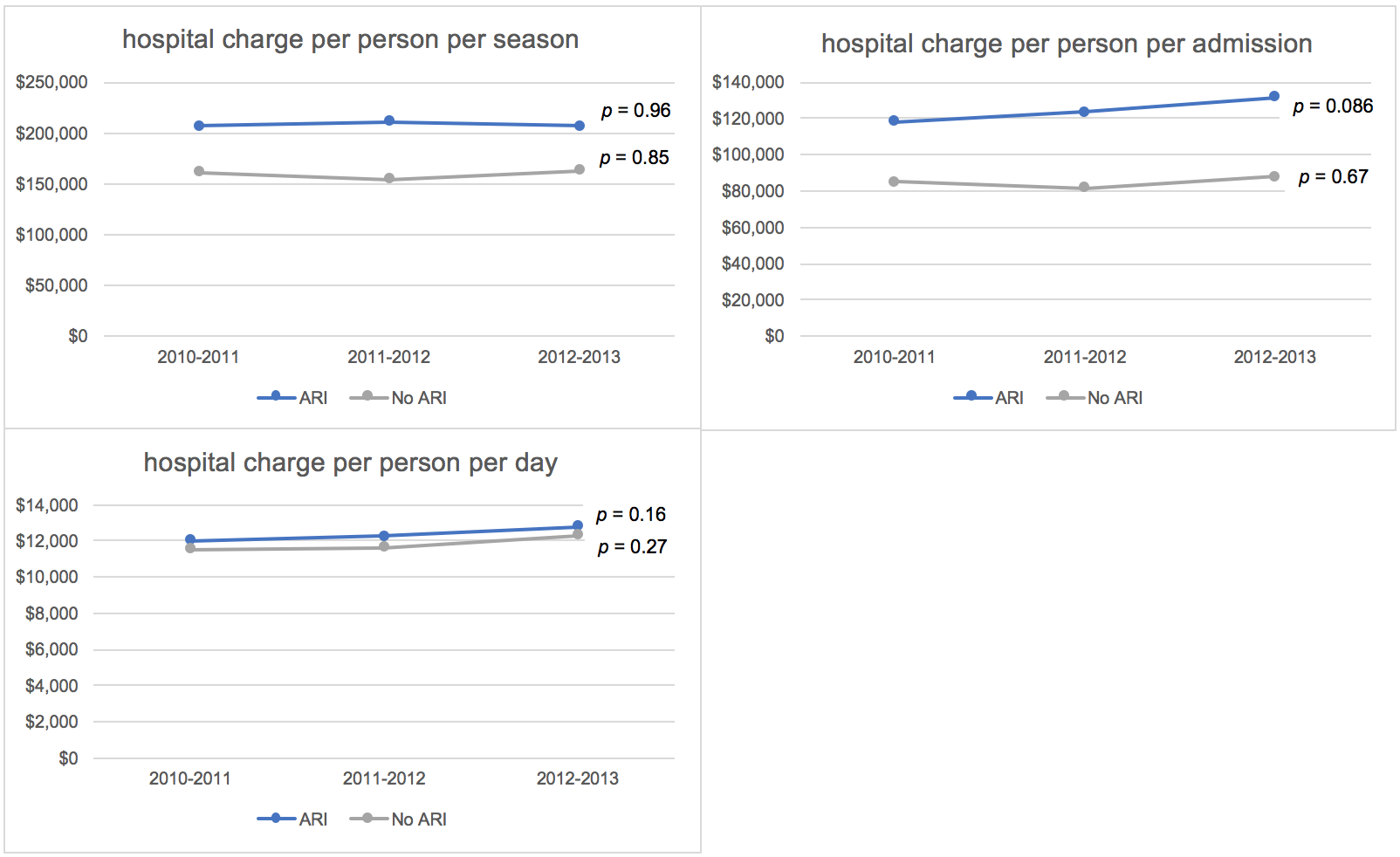


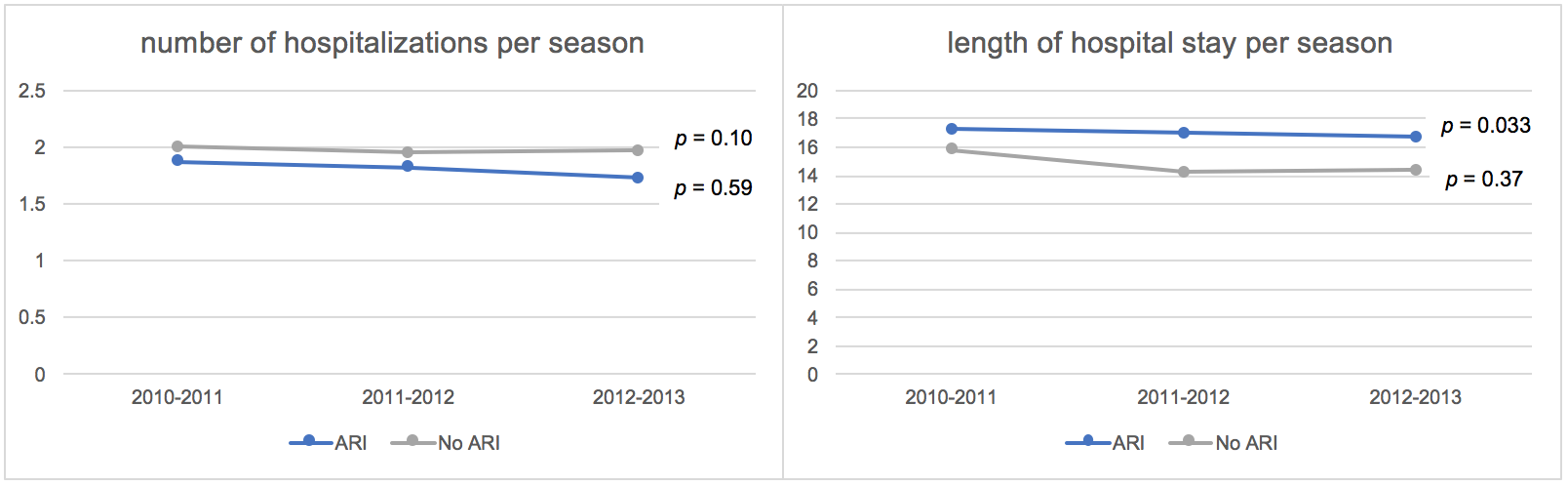


Supplementary Figure 2. Adjusted hospital charges per patient per season for cirrhotic patients with and without acute respiratory illness (ARI) based on mechanical ventilation in the 2011-2012 season


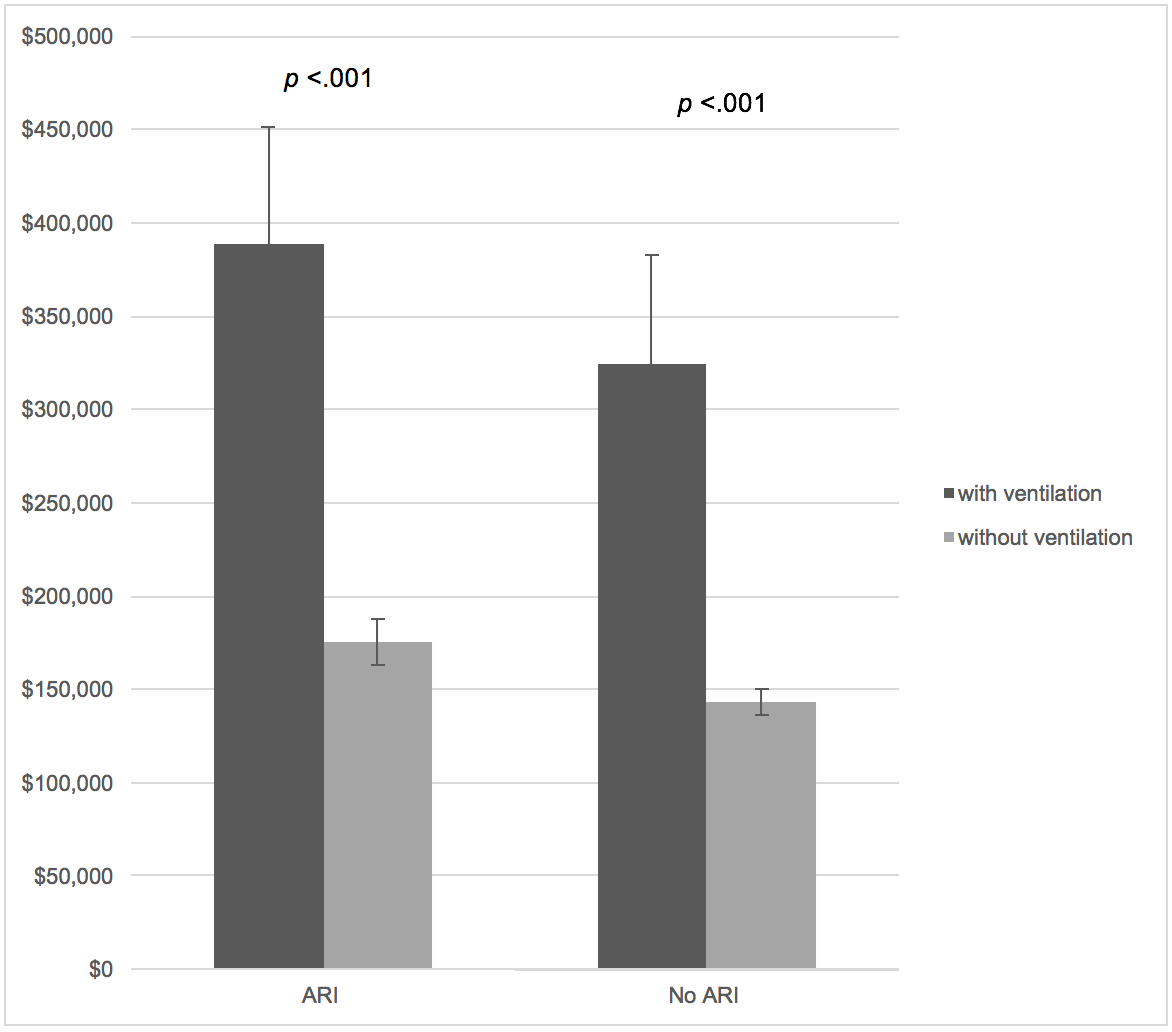


Results are adjusted for age, sex, race, ethnicity, insurance, severity of cirrhosis, cause of cirrhosis, cause of cirrhosis, comorbidities and history of severe comorbidities.

The p-values above the bars mean the difference between cirrhotic patients with and without acute respiratory illness is significant.

The black lines on the bars represent the corresponding 95% confidence intervals.
